# Disrupted Nodal and Hub Organization Account for Brain Network Abnormalities in Parkinson’s Disease

**DOI:** 10.3389/fnagi.2016.00259

**Published:** 2016-11-10

**Authors:** Yuko Koshimori, Sang-Soo Cho, Marion Criaud, Leigh Christopher, Mark Jacobs, Christine Ghadery, Sarah Coakeley, Madeleine Harris, Romina Mizrahi, Clement Hamani, Anthony E. Lang, Sylvain Houle, Antonio P. Strafella

**Affiliations:** ^1^Research Imaging Centre, Campbell Family Mental Health Research Institute, Centre for Addiction and Mental Health, University of Toronto, TorontoON, Canada; ^2^Division of Brain, Imaging and Behaviour – Systems Neuroscience, Krembil Research Institute, University Health Network, University of Toronto, TorontoON, Canada; ^3^Division of Neurosurgery, Toronto Western Hospital, University Health Network, University of Toronto, TorontoON, Canada; ^4^Morton and Gloria Shulman Movement Disorder Unit & E. J. Safra Parkinson Disease Program, Neurology Division, Department of Medicine, Toronto Western Hospital, University Health Network, University of Toronto, TorontoON, Canada

**Keywords:** Parkinson’s disease, bradykinesia, cognitive impairment, resting-state functional MRI (rsfMRI), brain network

## Abstract

The recent application of graph theory to brain networks promises to shed light on complex diseases such as Parkinson’s disease (PD). This study aimed to investigate functional changes in sensorimotor and cognitive networks in Parkinsonian patients, with a focus on inter- and intra-connectivity organization in the disease-associated nodal and hub regions using the graph theoretical analyses. Resting-state functional MRI data of a total of 65 participants, including 23 healthy controls (HCs) and 42 patients, were investigated in 120 nodes for local efficiency, betweenness centrality, and degree. Hub regions were identified in the HC and patient groups. We found nodal and hub changes in patients compared with HCs, including the right pre-supplementary motor area (SMA), left anterior insula, bilateral mid-insula, bilateral dorsolateral prefrontal cortex (DLPFC), and right caudate nucleus. In general, nodal regions within the sensorimotor network (i.e., right pre-SMA and right mid-insula) displayed weakened connectivity, with the former node associated with more severe bradykinesia, and impaired integration with default mode network regions. The left mid-insula also lost its hub properties in patients. Within the executive networks, the left anterior insular cortex lost its hub properties in patients, while a new hub region was identified in the right caudate nucleus, paralleled by an increased level of inter- and intra-connectivity in the bilateral DLPFC possibly representing compensatory mechanisms. These findings highlight the diffuse changes in nodal organization and regional hub disruption accounting for the distributed abnormalities across brain networks and the clinical manifestations of PD.

## Introduction

The clinical phenotypes of Parkinson’s disease (PD) include cardinal motor symptoms such as tremor, rigidity, bradykinesia, and loss of postural stability, along with a set of non-motor symptoms such as depression, sleep disturbances, autonomic dysfunction and cognitive impairment (Barnum and Tansey, 2012; Bonnet et al., 2012). Cognitive impairment is one of the most common non-motor symptoms in PD. It ranges from mild cognitive impairment in different cognitive domains (e.g., attention, executive, visuospatial, and memory) to dementia, which is thought to be derived from dysfunction of different neurotransmitter systems/brain networks (Gratwicke et al., 2015) associated with Lewy pathology and Alzheimer’s disease pathology in cortical and subcortical regions (Halliday et al., 2014). The recent graph theoretical approaches may be suitable to capture such neuropathology involving diffuse area of brain as well as complex cognitive function involving interactions of multiple regions and networks, as it allows for characterization of functional interactions such as integration and segregation on multiple levels such as nodes, modules, and entire brain as well as for identification of hub regions (Bullmore and Sporns, 2009), revealing more information about brain network changes compared with other methods such as seed-based analysis and independent component analysis.

The graph theoretical approaches have revealed that human brains show the following topological characteristics (Bullmore and Sporns, 2009, 2012). First, individual brain regions termed ‘nodes’ typically have disproportionate connections with other nodes. Highly connected nodes are considered as hubs, which play a crucial role in integrating information. The anterior and posterior cingulate cortices, insula, and superior frontal and parietal cortex are identified as both structural and functional hubs (van den Heuvel and Sporns, 2013; Sporns, 2014). These are heteromodal areas that are involved in a broad range of cognitive processes (Achard et al., 2006). Second, some nodes are more closely connected to one another and form modules, which promote specialized processing and functional segregation. Large-scale modules correspond to well-established resting-state functional networks (Engel et al., 2013; Stam, 2014). Third, these modules are also interconnected via hubs with long-distance pathways and thereby promote functional integration. Brain networks display small-world properties of efficient global and local parallel information transfer (i.e., integration and segregation) at relatively low connection cost (Achard and Bullmore, 2007), which reflects an optimal organization through evolution and development (Gong et al., 2009; Bullmore and Sporns, 2012). The coexistence and balance of segregation and integration of modules are fundamental for brain function (Bullmore and Sporns, 2012; Sporns, 2013).

There is evidence that the pathological course of neurodegenerative diseases, such as Alzheimer’s disease, target network hubs (Seeley et al., 2009; Stam et al., 2009), and selective damage to hubs can significantly change the integration of brain networks (Bullmore and Sporns, 2012). With the dysfunction of multiple neurotransmitters (Gratwicke et al., 2015; Pagonabarraga et al., 2015) and deposition of alpha-synuclein (Braak et al., 2005), network hubs are almost certainly vulnerable in PD (Stam, 2014), and the selective deterioration of these network hubs may account for the distributed abnormalities across the brain in PD (McColgan et al., 2015) with distinct clinical correlations.

The premotor area and pre-supplementary motor area (SMA) have been identified as key regions connecting multiple networks in healthy adults (Spreng et al., 2013). These regions are important for self-initiated movements and preparation for actions (Passingham et al., 2010). The SMA consisting of SMA proper and pre-SMA, is part of the basal ganglia thalamo-cortical motor circuit (Alexander et al., 1986) associated with dopamine and the changes in functional connectivity have been well documented in PD patients, along with other cortical motor areas such as the primary motor cortex and premotor area (Wu et al., 2009a).

The dorsolateral prefrontal cortex (DLPFC) has been implicated as an anatomical and a functional node associated with executive functions (Spreng et al., 2013; van den Heuvel and Sporns, 2013). This brain region is part of fronto-parietal network (FPN) or executive control network (Dosenbach et al., 2007; Seeley et al., 2007), as well as the fronto-striatal dopamine network (Gratwicke et al., 2015). PD patients often show executive dysfunction due to a disruption of the fronto-striatal dopamine system (Gratwicke et al., 2015). In addition, structural (Koshimori et al., 2015) and functional (Trujillo et al., 2015; Wu et al., 2015) changes in this cortical area and their association with cognitive impairment have been reported consistently in these patients (Koshimori et al., 2015).

Another important disease-associated hub in PD is the insular cortex, which connects and integrates several functional systems: the anterior area is involved in cognitive and behavioral/emotional functions, and the mid to posterior area involved in sensorimotor functions (Kurth et al., 2010). The insular cortex has extensive connections with the striatum in a connectivity gradient from posterior to anterior (Christopher et al., 2014a). In post-mortem PD brains, alpha-synuclein depositions become evident throughout the insula at Braak’s stage V (Braak et al., 2005). In addition, the level of CSF alpha-synuclein concentration was significantly associated with anterior insular network disruption (Madhyastha et al., 2015). The anterior insula is a core region of the salience network that guides behavior (Menon and Uddin, 2010), and this brain network can affect other cognitive networks such as the default mode network (DMN) (Bonnelle et al., 2012; Chiong et al., 2013). Graph theoretical analyses have revealed a decreased hub role in the left dorsal anterior insula in PD patients compared to healthy controls (HCs) (Tinaz et al., 2016). Furthermore, recent data from our group have demonstrated that D2 receptor availability in the anterior insula was associated with executive and memory dysfunction in PD patients with mild cognitive impairment (Christopher et al., 2014b, 2015). Thus, the anterior insula, in particular, seems to be a critical “hub” for cognitive impairment in Parkinsonian patients (Christopher et al., 2014a; Criaud et al., 2016).

The present study aimed to investigate whether nodal connections and network hubs were affected in PD patients, and whether these changes were associated with motor and non-motor symptoms employing graph theoretical approaches to resting-state functional MRI (rsfMRI) data. We hypothesized that (1) PD would affect the nodal and hub organization of regions such as the SMA, insula, DLPFC, as well as the striatum, and (2) the nodal and hub changes would account for some of the motor and cognitive symptoms of PD.

## Materials and Methods

### Participants

Forty-five patients meeting the UK Brain Bank criteria for the diagnosis of idiopathic PD and 25 HCs participated in the study. During the screening session, all participants provided written informed consent following a full explanation of the study procedures. Subsequently, they were assessed for their global cognitive performance using the Montreal Cognitive Assessment (MoCA) and their level of depression using the Beck Depression Inventory (BDI) II. Additionally, patients were assessed for their motor severity of the disease using the motor subset of the Unified Parkinson Disease Rating Scale (UPDRS) III and for disease stage using the Hoehn and Yahr. The Levodopa Equivalent Daily Dose (LEDD) was also calculated by Levodopa (mg/day) + Controlled Levodopa (Levodopa × 0.75 mg/day) + Entacapone/Comtan (Levodopa × 0.33 mg/day) + Pramipexole/Mirapex (mg/day) + Ropinirole/Requiq × 20 (mg/day) + Rasagiline/Azilect × 100 (mg/day) + Selegiline × 10 (mg) + Amantadine (mg/day) (Tomlinson et al., 2010). All the participants underwent a structural and rsfMRI scan. Patients underwent the study procedures in an on-medication state. We reasoned that (1) scanning patients in an on-medication state would minimize participants’ motion effects on rsfMRI data (Tahmasian et al., 2015), and (2) studying chronically medicated patients would permit us to capture the complex neural substrate of cognitive impairment because cognitive impairment in patients is mostly likely to be derived from dysfunction of multiple neurotransmitters (Gratwicke et al., 2015). Exclusion criteria for the participants included: (1) history of a head injury, psychiatric or neurological diseases (except PD for the patients), (2) alcohol or drug dependency or abuse, (3) contraindications for MRI scanning, (4) dyskinesias and dystonia for PD patients, and (5) for HCs, MoCA scores <26 and BDI II scores >13. The study was approved by the Centre for Addiction and Mental Health Research Ethics Board.

### MRI Image Acquisition

MR images were acquired with a General Electric Discovery MR 750 3T scanner with 8-channel head coil. The protocol included whole-brain anatomic T1-weighted MRI images (Fast Spoiled Gradient Echo pulse sequence; 200 sagittal slices; matrix of 256 × 256; Repetition Time: 6.7 ms; Echo Time: 3.0 ms; slice thickness: 0.9 mm; Field of View: 23 cm; Inversion Time: 650 ms; flip angle = 8°) and rsfMRI images (Gradient Echo/Fast Gradient Echo pulse sequence; 31 axial slices; matrix of 64 × 64; Repetition Time: 2000 ms; Echo Time: 30 ms; flip angle: 60°; Field of View: 22 cm; slice thickness: 5 mm; duration: approximately 8 min). During the rsfMRI scan, the participants were instructed to keep their eyes open, let their mind wander, not to think about anything in particular, and not to fall asleep. The first two volumes of the rsfMRI images were removed to allow for magnetization equilibrium, resulting in the preprocessing of 240 volumes.

### rsfMRI Preprocessing

The functional data were preprocessed for each subject using the *Conn* functional connectivity toolbox version 15a (Whitfield-Gabrieli and Nieto-Castanon, 2012) including slice timing correction, motion correction using a 6° rigid spatial transformation, which provided the spatial deviation for each time point for translational (*x, y, z*) and rotational (roll, pitch, yaw) directions of movement, normalization into standard Montreal Neurological Institute space using non-linear transformations, and smoothing with a Gaussian smoothing kernel of 6 mm full-width half maximum. Outliers in the global signal of brain activation and movement were examined using the artifact detection toolbox. Time points were considered as outliers if the global signal of brain activation exceeded three standard deviations of the mean or if movement exceeded 0.5 mm across translational and rotational directions of scan-to-scan deviation. Participants were excluded from the analyses if outliers accounted for greater than 20% of the entire dataset. Among the 70 participants, five participants (two HCs and three patients) were excluded due to excessive head motion and artifacts, resulting in 23 HCs and 42 patients included in the graph theoretical analysis. Realignment parameters for head motion including *x, y, z*, roll, pitch, yaw as well as the total number of outliers did not show significant group differences between HCs and patients. Nuisance signals including six motion parameters as well as the signal from white matter and CSF voxels were regressed using aCompCor, the component–based noise correction method (Behzadi et al., 2007). The residual datasets were then temporally filtered (0.008 < *f* < 0.09).

### Network Nodes

**FIGURE 1:**
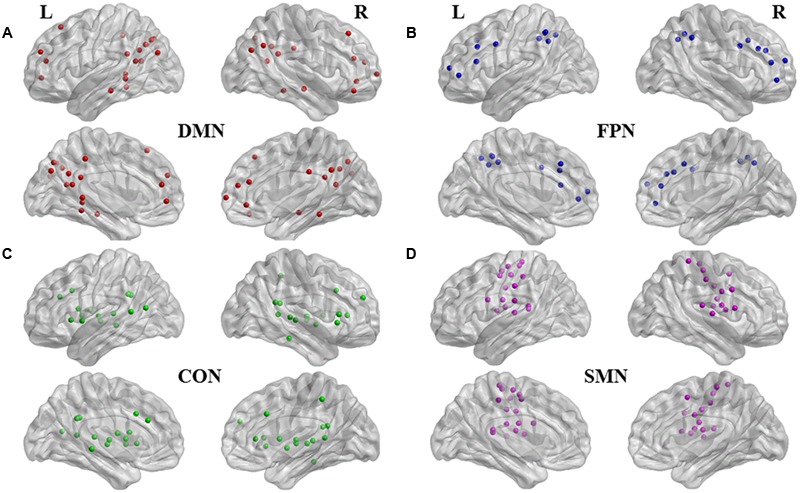
**Functional nodes investigated.** Medial and lateral views of brain images with 120 regions of interest of four subnetworks (Dosenbach et al., 2010). **(A)** 34 nodes in red are affiliated with default mode network (DMN); **(B)** 21 nodes in blue, with fronto-parietal network (FPN); **(C)** 32 nodes in green, with cinculo-opercular network (CON); and **(D)** 33 nodes in purple, with sensorimotor network (SMN). Brain image and nodes are visualized using the BrainNet Viewer (NKLCNL, Beijing Normal University).

Among 160 regions of interest of six different neural networks, derived from a series of five meta-analyses of fMRI activation studies combined with cognitive control nodes identified during error-processing (Dosenbach et al., 2010), we selected 120 regions of interest in spheres with a radius of 5 mm in four networks (**Figures 1A–D**) to investigate the hub regions associated with motor and cognitive symptoms of PD. These included 34 nodes of the DMN, 21 nodes of the FPN, 32 nodes of the cingulo-opercular network (CON), and 33 nodes of the sensorimotor network (SMN).

### Computation of Network Measures

Network measures were computed with age as a covariate using a Graph-Theoretical Analysis Toolbox (Hosseini et al., 2012). This toolbox constructs a binary undirected graph G that has a network degree of E equal to the number of edges, and a network density (cost) of *D* = *E*/[N × (*N*-1)]/2 representing the ratio of existing edges relative to all possible edges (Bruno et al., 2012). It also identifies network fragment. Network measures were computed over a range of connection densities. We examined those graphs where all nodes were fully connected in the networks for both groups, which ensured that network comparison between groups was meaningful with the same number of nodes in each module, and where small-world properties were displayed for both groups. Network fragment was observed at the density of 40%. Animal research has indicated that densities above 50% are unlikely to be biological (Kaiser and Hilgetag, 2006). Therefore, the densities ranging from 41 to 50% with an increment of 1% were investigated. Both HCs and patients displayed small worldness properties where normalized characteristic path length is close to one and normalized clustering coefficient is greater than one. Our interest was to detect changes in nodal characteristics including hubness. Therefore, we investigated the graph measures of local efficiency, as well as of nodal degree and betweenness centrality (BC) (Bullmore and Sporns, 2009; Rubinov and Sporns, 2010). Local efficiency measures the efficiency of local communication of a given node, taking account for the number of the shortest paths between its neighboring nodes (Latora and Marchiori, 2001; Rubinov and Sporns, 2010). Nodal degree is defined as the total number of connections that a node has with other nodes in the network, and BC is defined as the fraction of all shortest paths in the network that pass through a given node. Bridging nodes that connect disparate parts of the network often have high BC, making a potential hub role of these nodes in the network (van den Heuvel and Hulshoff Pol, 2010), that interact with many other regions and facilitate functional integration (Rubinov and Sporns, 2010).

### Seed-Based Functional Connectivity Analysis

The only nodes showing group differences were further investigated with seed-based functional connectivity analyses to elucidate changes in connectivity with other nodes in the network using the *Conn* functional connectivity toolbox version 15a.

### Statistical Analysis

Statistical analyses for demographic measures were performed using SPSS Statistics 20.0.0^1^ (Chicago, IL, USA). Statistical significance threshold was set at *P* ≤ 0.05. Independent student’s *t*-test was used to compare the means in head motion parameters and demographic measures between patients and HCs.

Area under the curve (AUC) analyses were performed to test group differences in graph measures using a two-tailed non-parametric permutation test with 1000 repetitions over the density range without network fragmentation using Graph-Theoretical Analysis Toolbox. The AUC analyses are less sensitive for thresholding processes as they yield a summarized scalar independent of single threshold selection. Statistical significance threshold was set at *P* ≤ 0.005.

As a hub is characterized both by high connectivity and centrality (Sporns et al., 2007), the nodes whose degree and BC were one standard deviation above the mean network degree and BC were considered hubs. Spearman correlation analyses were also performed between the only network measures that yielded significant group differences, and different disease measures (UPDRS III total scores, rigidity, bradykinesia, and resting tremor sub-scores, LEDD, MoCA and BDI II scores) using SPSS Statistics 20.0.0^2^ (Chicago, IL, USA) Statistical significance threshold was set at *P* ≤ 0.05. General linear model was used to obtain between-subjects contrasts of seed-based functional connectivity analysis. Statistical significance threshold was set at *P* ≤ 0.005.

## Results

### Demographic and Clinical Characteristics

**Table 1** shows the demographic and clinical characteristics of the patients and HCs. There were significant differences in MoCA (*t* = 3.216, *P* = 0.002), and BDI II scores (*t* = -4.27, *P* < 0.001) between the two groups. PD patients showed lower global cognitive performance and higher depression level than HCs. There was no significant difference regarding age (*t* = 0.588, *P* = 0.56), or education (*t* = 0.179, *P* = 0.86) between the two groups.

**Table 1:** Demographic and clinical characteristics of patients with Parkinson’s disease (PD) and healthy controls (HCs).

	Patients (*n* = 42)	HCs (*n* = 23)
Age (years)	65.4 (6.5)	64.3 (8.3)
Sex (male/female)	31/11	10/13
Handedness (right/left)	34/8	23/0
Education (years)	16.0 (3.2)	16.1 (3.1)
Montreal Cognitive Assessment (MoCA)	25.8 (2.9)^∗^	27.5 (1.4)
Beck Depression Inventory II (BDI)	8.0 (4.6) ^∗∗^	3.2 (3.4)
Disease duration (years)	5.9 (4.5)	-
Symptom-dominant side (right/left)	26/16	-
Unified Parkinson’s Disease Rating Scale III (UPDRS) (on-medication)	27.5 (10.1)	-
Rigidity	5.1 (2.2)	-
Bradykinesia	1.9 (0.6)	-
Resting tremor	1.3 (1.3)	-
Hoehn and Yarhr	2 (median)	-
Levodopa Equivalent Daily Dose (LEDD) (mg/day)	644.4 (298.5)	-

*Data are presented in mean (standard deviation) unless otherwise indicated; ^∗^*P* < 0.01; ^∗∗^*P* < 0.001.*

### Group Comparisons of Graph Measures

**FIGURE 2:**
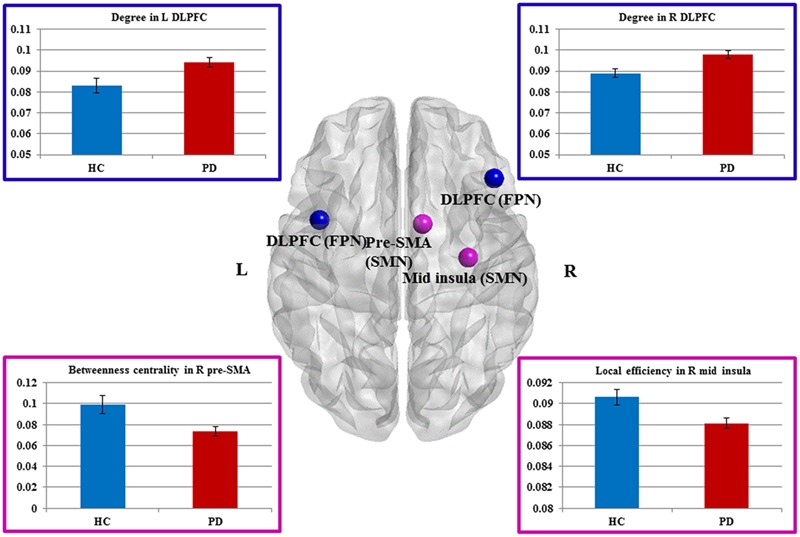
**Sensorimotor and fronto-parietal nodes showing group differences.** Dorsal view of brain image presenting four nodes showing group differences. Increased degree in the bilateral DLPFC of FPN, as well as decreased betweeness centrality in the right pre-SMA and decreased local efficiency in the right mid-insula of SMN in Parkinson’s disease (PD) patients compared with healthy controls (HCs) (*P* ≤ 0.005, uncorrected). The nodes are presented in equal size for visualization purposes. Brain image and nodes are visualized using the BrainNet Viewer (NKLCNL, Beijing Normal University). Bar graphs show the averaged values of each graph measure for HCs and PD (patients with Parkinson’s disease). Error bars represent standard error of the mean.

Compared to HCs, patients showed increased numbers of connections in the right and left DLPFC measured by nodal degree (*P* = 0.005 and *P* = 0.003, respectively) (**Table 2**; **Figure 2**). In contrast, patients showed a reduction in intra-connectivity of the right mid-insula measured by local efficiency (*P* = 0.005), as well as a reduction in bridging role of the right pre-SMA (*P* = 0.005) measured by nodal BC.

**Table 2:** Graph measure changes in patients with PD compared with HCs.

	Graph measure	Node	Subnetwork	BA	Coordinate#		
					*x*	*y*	*z*
HCs < patients	Degree	Right DLPFC	Fronto-parietal network (FPN)	9	46	28	31
		Left DLPFC	FPN	9	-42	7	36
HCs > patients	Local efficiency	Right mid-insula	Sensorimotor network (SMN)	-	33	-12	16
	BC	Right pre-SMA	SMN	6	10	5	51

*BA, Broadmann Area; DLPFC, dorsolateral prefrontal cortex; pre-SMA, pre-supplementary motor cortex; #the Montreal Neurological Institute coordinates describe the center of node area.*

We interrogated whether the graph measures in these four nodes would account for any of the clinical measures. We found that BC of the right pre-SMA showed a significant negative correlation with bradykinesia scores (*r* = -0.31, *P* = 0.048), suggesting that the diminished bridging role (i.e., impaired integration) of the right pre-SMA may contribute to more severe bradykinesia. In addition, we conducted exploratory correlation analyses between the bilateral DLPFC and MoCA subscores for executive function and attention because the DLPFC is involved in both functions. Although we did not find any correlations in PD patients, interestingly, PD patients performed as well as HCs on these subtests.

We then applied the seed-based functional connectivity analyses to explore how these four affected areas changed their connectivity with other nodes of the network. The results are summarized in **Table 3**. First, nodes in the DLPFC of FPN showed increased intra-connectivity with one another. This increased regional inter-connectivity may occur as a compensatory mechanism. Second, the right pre-SMA of the SMN showed reduced inter-connectivity with nodes in the DMN, suggesting its bridging and integrative role between cognitive and motor functions.

**Table 3:** Functional connectivity of three nodes that showed group differences.

	Graph measure	Seed region	Subnetwork	Coordinate#			Target region	Subnetwork	Coordinate#			*T* (63)	*P* (uncorrected)
				*x*	*y*	*z*			*x*	*y*	*z*		
HCs < Patients	Degree	Right DLPFC (BA9)	FPN	46	28	31	Right mid-insula	Cingulo-opercular network (CON)	32	-12	2	4.01	<0.001
							Left posterior insula	CON	-30	-28	9	3.23	<0.001
							Right DLPFC (BA9)	FPN	40	17	40	2.95	0.002
							Left putamen	CON	-20	6	7	2.7	0.004
		Left DLPFC (BA9)	FPN	-42	7	36	Right DLPFC (BA9)	FPN	40	17	40	2.68	<0.005
HCs > Patients	BC	Right pre-SMA (BA6)	SMN	10	5	51	Left angular gyrus (BA40)	Default mode network (DMN)	-48	-63	35	3.29	<0.001
							Left inferior temporal cortex (BA20)	DMN	-59	-25	-15	2.81	0.003

*BA, Broadmann Area; DLPFC, dorsolateral prefrontal cortex; pre-SMA, pre-supplementary motor area; # the Montreal Neurological Institute coordinates describe the center of node area.*

### Hub Analysis

**FIGURE 3:**
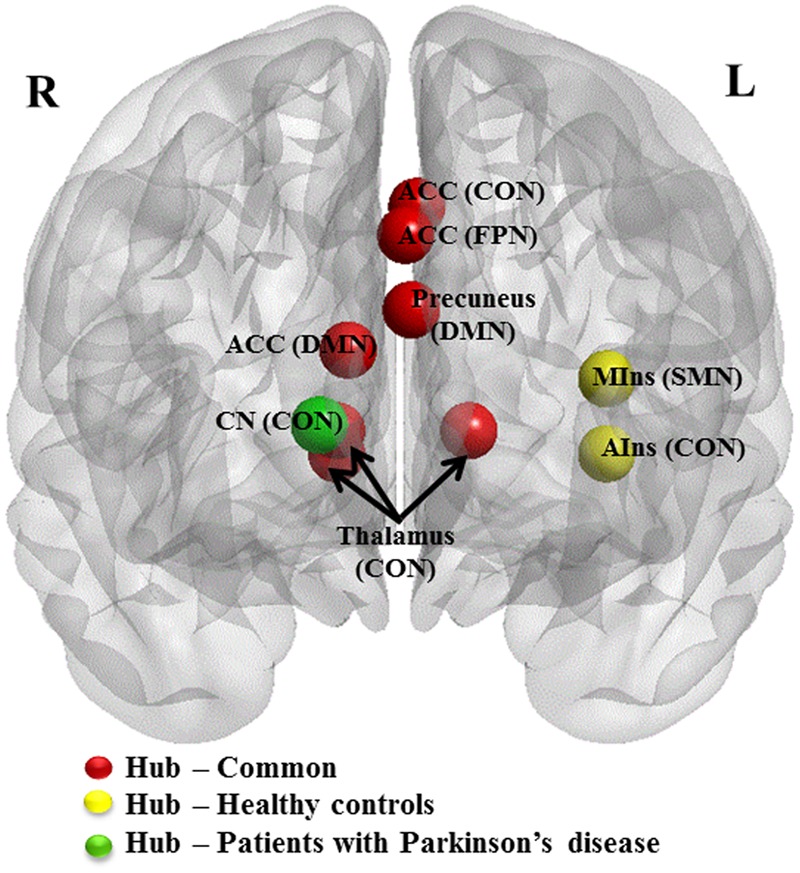
**Hub regions in patients with PD and HCs.** Caudal view of brain image presenting 10 hub regions. ACC, anterior cingulate cortex; AIns, anterior insula; CN, caudate nuculeus; CON, cingulo-opercular network; DMN, default mode network; FPN, fronto-parietal network; MIns, mid-insula. Seven hubs in red represent common hubs to both HCs and patients with PD. Two in yellow represent hubs identified only in HCs and one in green, only in patients. Brain image and hubs are visualized using the BrainNet Viewer (NKLCNL, Beijing Normal University).

Patients presented eight regions acting as hubs while HCs presented nine regions (**Table 4**). Seven of these regions were shared by both patients and HCs. The patients lost hub properties in the left anterior insula of the CON and the left mid-insula of the SMN, while the right caudate nucleus of the CON acted as a new hub in the patients (**Figure 3**). We explored whether any of these three hub regions were associated with the clinical measures. We found a significant positive correlation between hubness (i.e., mean of degree and BC) in the left mid-insula and dopaminergic medication (*r* = 0.3, *P* = 0.05), suggesting that dopaminergic medication may influence hubness in the mid-insula in these patients. We conducted further exploratory correlation analyses between the left anterior insula and the right caudate nucleus, and executive function and memory as the insula was associated with both executive and memory function in PD patients. No correlations were found in PD patients. However, PD patients performed significantly worse than HCs on the memory subtest (PD: 2.9 ± 1.4 and HC: 3.7 ± 1.3, *t* = 2.1, *P* = 0.038).

**Table 4:** Hub regions in patients with PD and HCs.

	Hub region	Subnetwork	Coordinate#		
			*x*	*y*	*z*
Common	Right anterior cingulate cortex	DMN	9	39	20
	Left precuneus	DMN	-3	-38	45
	Left anterior cingulate cortex	FPN	-1	28	40
	Left anterior cingulate cortex	CON	-2	30	27
	Left thalamus	CON	-12	-12	6
	Right thalamus	CON	11	-12	6
	Right thalamus	CON	11	-24	2
HC only	Left anterior insula	CON	-36	18	2
	Left mid-insula	SMN	-36	-12	15
Patient only	Right caudate nucleus	CON	14	6	7

*#The Montreal Neurological Institute coordinates describe the center of node area.*

## Discussion

In the current study, we aimed to investigate functional changes in the SMN and cognitive networks including the DMN, FPN, and CON in PD with a particular focus on changes in the disease-associated nodes and hubs using graph theoretical analyses. In general, patients showed weakened connectivity in nodes of the SMN and enhanced connectivity in nodes associated with cognitive networks. Patients showed functional changes in the right pre-SMA and right mid-insula, as well as the bilateral DLPFC (BA9) compared with HCs. PD also affected hub functions and organization. In particular, the left anterior and left mid-insula cortex lost their hub properties and a node in the caudate nucleus was identified as a new hub in these patients.

### Changes in the SMN

As hypothesized, we found functional changes in nodes of the SMN, which is consistent with previous studies using the graph theoretical approaches (Wu et al., 2009b; Skidmore et al., 2011; Nagano-Saito et al., 2014; Wei et al., 2014; Tinaz et al., 2016). Specifically, patients showed a reduction in intra-connectivity of the right mid-insula and impaired integration of the right pre-SMA with nodes of the DMN. The diminished bridging role of the right pre-SMA was associated with more severe bradykinesia. The left mid-insula lost its hub properties in the patients and showed a positive interaction with dopaminergic medication.

The pre-SMA is the anterior part of the SMA and is separated from the SMA proper by the vertical anterior commissural line (Picard and Strick, 2001). These two areas are different in terms of anatomical connections and functions. The SMA proper is connected to the primary motor cortex, projected to the spinal cord and is involved in movement generation, while the pre-SMA has extensive connections with the prefrontal cortex (Wang et al., 2001) and is involved in cognition (Picard and Strick, 2001). For example, the pre-SMA has been implicated to play an important role in cognitive motor control involving integration of sensory information and decision-making about actions or motor selection (Ikeda et al., 1999).

It is not surprising that the decreased inter-connectivity of the pre-SMA with a cognitive network can account for the slowness of movement, as it is postulated that bradykinesia could arise from slowness in formulating the instructions to move before the onset of and during the actions (Berardelli et al., 2001). As the right pre-SMA, but not the left pre-SMA has functional connectivity with the DMN (Ter Minassian et al., 2014), the weakened connectivity between the right pre-SMA and the nodes of the DMN, in the context of dopamine deficiency, may affect the redirection of attentional processes from self-reflection (i.e., internal reference) to goal-directed behavior, thus contributing to bradykinesia.

We found that the left mid-insula correlated with LEDD, but not with the UPDRS motor scores or subscores. This might be because the mid-insula is implicated in the processing of position, movement, and sensation of the body (Cerasa et al., 2006; Chang et al., 2013). The UPDRS-III and the motor subscores may not closely reflect the function of mid-insula when patients are in an on medication state. In addition, the presence or absence of correlation between the right and left mid-insula of SMN and LEDD might be due to the differences in their network measures (i.e., local efficiency vs. hubness).

### Changes in the Cognitive Networks

We also found functional changes in the DLPFC (BA9) associated with the FPN and the left anterior insula of the CON in the patients compared with HCs. The patients showed an increased level of connectivity in bilateral DLPFC (BA9). The subsequent functional connectivity analysis revealed that the enhanced connectivity occurred within and between cognitive networks (i.e., FPN and CON). The hub analysis revealed that while the left anterior insula lost its hub properties, a new hub region was identified in the striatal region (i.e., caudate nucleus) associated with the same network (i.e., CON) in the Parkinsonian patients.

In aging brains, hyper-activations are interpreted as compensatory responses when older adults perform at the same level as younger adults or when increased brain activity is positively correlated with cognitive performance in older adults, but not in younger adults (Grady, 2012). Our patients had significantly lower MoCA scores compared with HCs, and there were no positive correlations between the network measures and MoCA scores, suggesting that the enhanced connectivity of the bilateral DLPFC and the new hubness of the caudate nucleus may be parallel “attempted compensation” (Grady, 2012) for trying to maintain an efficient utilization of neural resources (Grady, 2008) in the context of dopamine deficiency. On the other hand, the fact that MoCA subscores for executive function and attention (Nasreddine et al., 2005) in PD patients were not significantly different from those in HCs suggests that the enhanced connectivity may be successful instead of “attempted” compensatory responses for the particular cognitive functions.

The emergence of the right caudate nucleus as a new hub may be explained by the “hub failure” (Stam, 2014) of the left anterior insula. This model postulates that affected hubs reroute their connections to nodes at lower levels of hierarchy, resulting in the local emergence of new connections, which may also be an emergence of new hubs in the same network as observed in our data. The underpinning of the left lateralization of the affected anterior insular is unknown. However, the verbal memory dysfunction (Christopher et al., 2015) in our patients might be associated with the left lateralization. Because of the extensive connections between the anterior insula and the caudate nucleus, the deteriorating left anterior insula may have rerouted some of the connections to the right caudate nucleus.

### Dopaminergic Modulation on Resting-State Functional Connectivity

Our PD patients underwent all study procedures in an on medication state in order to minimize the motion effects on the rsfMRI data. However, we acknowledge the confounding effects of dopaminergic medication on rsfMRI in PD patients. Dopamine replacement therapy typically normalizes aberrant functional connectivity patterns in sensorimotor nodes and network (Tahmasian et al., 2015) although there are differential effects of the medication on the normalization (Wu et al., 2009b). On the other hand, dopaminergic medications can have differential effects on cognitive nodes and networks. Our findings of reduced functional connectivity in the SMN nodes including the pre-SMA and mid-insula likely reflected the abnormalities that were not completely restored by dopaminergic medication while other more overt sensorimotor nodes were normalized. In addition, the pre-SMA that involves in cognitive control for action may be affected by dopamine replacement therapy and dopamine agonists differently from other sensorimotor nodes.

The failing hub function of the left anterior insula in the medicated patients suggests contribution of other pathology and/or inadequate effects of dopaminergic medication. Increased functional connectivity in the bilateral DLPFC that were likely compensatory responses may be associated with the on medication state as functional connectivity in the DLPFC is generally affected by dopaminergic medication. However, we did not find the association between the brain changes and LEDD. Further studies are needed to investigate the modulating effects of dopaminergic medication on network measures by contrasting them in the same cohort of PD patients in on and off medication states.

### Heterogeneity of PD Patients

PD patients in the current study were clinically heterogeneous. Therefore, our findings may have reflected functional changes derived from those with certain clinical and cognitive/behavioral characteristics. For example, 13 of the patients (31%) had MoCA scores lower than 26. PD patients had significantly higher BDI II scores than HCs although only three patients had the scores higher than 13, and the depression levels of our patients were below the cur-off scores on average. The duration of disease ranges from 1 to 18 years. There were one patient at Hoehn and Yarhr stage 1, nine at stage 1.5, twenty-seven at stage 2, four at stage 2.5, and one at stage 3. We did not find any correlations between the graph measures and these clinical and cognitive/behavioral measures. However, further studies are needed to elucidate specific network changes in PD with clinical and cognitive/behavioral subtypes.

## Summary and Conclusion

In summary, these findings highlight the diffuse changes in the nodal organization along with the regional hub disruption, which accounts for the distributed abnormalities across brain networks and clinical manifestations of PD. Some of these network alterations may be adaptive and counteracting the influence of neurotransmitter dysfunction and deposition of alpha-synuclein, resulting in a blurring of network boundaries required for distinct cerebral functions.

## Author Contributions

YK, S-SC, and AS: study conception and design; YK: data acquisition: YK, S-SC, MC; data analysis; YK, S-SC, LC, MJ, CG, SC, MH, RM, CH, AL, SH, and AS: date interpretation; YK: manuscript drafting; S-SC, LC, MJ, CG, SC, MH, RM, CH, AL, SH, and AS: manuscript review and critique; YK, S-SC, LC, MJ, CG, SC, MH, RM, CH, AL, SH, and AS: manuscript final approval.

## Conflict of Interest Statement

The authors declare that the research was conducted in the absence of any commercial or financial relationships that could be construed as a potential conflict of interest.

## Footnotes

1 http://www-01.ibm.com/software/analytics/spss/

2 http://www-01.ibm.com/software/analytics/spss/
